# Gulf War Illness-associated increases in blood levels of interleukin 6 and C-reactive protein: biomarker evidence of inflammation

**DOI:** 10.1186/s13104-019-4855-2

**Published:** 2019-12-18

**Authors:** Tammy A. Butterick, Janeen H. Trembley, Laura L. Hocum Stone, Clemma J. Muller, Rebecca R. Rudquist, Ronald R. Bach

**Affiliations:** 1 Minneapolis Veterans Affairs Health Care System, Minneapolis, MN USA; 2 Department of Food Science and Nutrition, University of Minnesota, St Paul, MN USA; 3 Masonic Cancer Center, University of Minnesota, Minneapolis, MN USA; 4 Department of Laboratory Medicine and Pathology, University of Minnesota, Minneapolis, MN USA; 5 Department of Surgery, University of Minnesota, Minneapolis, MN 55455 USA; 6 Elson S Floyd College of Medicine, Washington State University, Spokane, WA USA; 7 Department of Medicine, University of Minnesota, Minneapolis, MN USA

**Keywords:** Gulf War Illness, Chronic multisymptom illness, Chronic inflammation, Biomarkers, Proinflammatory cytokines, Interleukin 6, C-reactive protein, Case–control study

## Abstract

**Objective:**

Gulf War Illness is a chronic multisymptom disorder severely impacting the health and well-being of many Veterans of the 1990–1991 Gulf War. Symptoms that define the disease include pain, fatigue, mood and memory impairments, gastrointestinal problems, lung disorders, and skin rashes. In our previous biomarker study, we discovered Gulf War Illness-associated proinflammatory blood biomarkers. Therefore, we hypothesized that chronic inflammation causes the symptoms that define this disorder. Testing the chronic inflammation hypothesis is the objective of this study.

**Results:**

The biomarker fingerprint of Gulf War Illness is the end-product of a cascade of proinflammatory cytokine signals. In particular, the observed increase in C-reactive protein predicts a corresponding increase in interleukin 6, the cytokine that stimulates hepatocytes to produce C-reactive protein. Therefore, in this study we measured potential upstream cytokine signals in plasma samples from Gulf War Veterans. As predicted, a positive correlation between interleukin 6 and C-reactive protein was observed.

## Introduction

From August 2, 1990 to July 31, 1991, nearly 700,000 US military personnel served in the First Gulf War, Operations Desert Shield and Desert Storm. Now, many of these Gulf War Veterans suffer from an unexplained chronic multisymptom illness (CMI). A 2014 report from National Academy of Sciences Institute of Medicine to the Department of Veterans Affairs (VA) recommended the name should be changed from CMI to Gulf War illness (GWI) [1]. Another recommendation in the same report was the use of either the Kansas or the Centers for Disease Control and Prevention (CDC) case definitions for GWI [2, 3]. They noted the symptomatic criteria defining this syndrome are well characterized by both case definitions. There are six GWI-associated symptom domains: (1) fatigue/sleep problems, (2) pain (musculoskeletal), (3) mood-cognition, (4) gastrointestinal, (5) pulmonary, and (6) skin (rashes). The high prevalence of these symptoms amongst Gulf War Veterans makes GWI the signature adverse health-related outcome of the 1990–1991 Gulf War [2–6].

The accurate diagnosis and effective treatment of GWI require a detailed understanding of the underlying disease. The lack of information about GWI pathophysiology led us to initiate a search for objective biomarkers to augment current symptomatic diagnostic criteria. In our case–control observational study we identified 11 blood biomarker differences between symptomatic (GWI+) and asymptomatic (GWI−) Gulf War Veterans [7].

Plasma proteomics identified 6 biomarker differences., and blood cell enumeration identified 5 biomarker differences. All 11 GWI-associated blood biomarker differences are potential indicators of inflammation. In particular, there was the GWI-associated increase in C-reactive protein (CRP). CRP is a well-established biomarker of inflammation produced by hepatocytes. A pro-inflammatory cytokine cascade involving both interleukin 1 beta (IL-1β) and interleukin 6 (IL-6) is the signal stimulating CRP gene expression. The initial biomarker study did not yield useful data for either IL-1β or IL-6. We hypothesized that a more sensitive cytokine immunoassay would reveal GWI-associated proinflammatory cytokine increases. This evidence would support our hypothesis that chronic inflammation is the underlying cause of GWI-associated symptoms.

## Main text

### Methods

In the current study, pro-inflammatory cytokine levels were measured in plasma samples from the parent study entitled “Biomarkers of Gulf War Veterans’ Illnesses: Tissue Factor, Chronic Coagulopathy, and Inflammation.” Gulf War Veterans entered the case–control observational human study between 2010 and 2013. The Veterans were interviewed in person, and written informed consent was obtained. Health status was assessed via a structured interview, and blood samples were obtained.

The only inclusion criteria for Veterans enrolled in this study were honorable discharge from US military service and deployment to the Kuwaiti Theater of Operations during the 1990–1991 Gulf War. Exclusion criteria included history of cancer, liver disease, acute or chronic inflammatory states, or other major chronic illness such as chronic fatigue syndrome and fibromyalgia that could be associated with inflammation. Post-traumatic stress disorder did not exclude subjects.

All subjects completed a symptom questionnaire, the CDC 10 question symptoms assessment survey (CDC-10), for CMI developed by Fukuda et al. [3]. This survey included three categories and nine subcategories: (1) fatigue; (2) mood-cognition (depression, anxiety, moodiness, memory problems, difficulty with words, difficulty sleeping); and (3) musculoskeletal (muscle pain, joint pain, and joint stiffness). Subjects were considered to have GWI and were classified as GWI+ if: (a) they had one or more chronic symptoms from at least 2 of 3 of the case-defining symptom categories—fatigue, mood-cognition, and musculoskeletal pain; (b) the symptoms began during or after the 1990–1991 Gulf War; and (c) symptoms were present for at least 6 months. Subjects without case-defining symptoms or a symptom in only one category were considered not to have GWI and were classified as GWI−.

Plasma samples were obtained as described [7]. Briefly, non-fasting, peripheral venous blood was collected into 4.0 ml Vacutainer1 vacuum tubes (BD, Franklin Lakes, NJ, USA) containing 7.2 mg K2 EDTA. Platelet-poor plasma was isolated from whole blood by centrifugation at 1770×*g* for 15 min at room temperature. The plasma layer was carefully removed and centrifuged at 1770×*g* for 15 min until the platelet count was ≤ 1/μl (Beckman Coulter AcTdiff 2 counter, Brea, CA, USA). Plasma aliquots were snap frozen on dry ice and stored at − 80 °C.

We employed the Meso Scale Discovery (MSD) (Rockville, MD, USA) plate-based electrochemiluminescence (ECL) assay platform to quantify plasma concentrations of interferon gamma (IFN-γ), IL-1β, interleukin 2 (IL-2), interleukin 4 (IL-4), IL-6, interleukin 8 (IL-8), interleukin 10 (IL-10), interleukin 12 p70 (IL-12 p70), interleukin 13 (IL-13), and tumor necrosis factor alpha (TNF-α). Stored plasmas from the initial GWI biomarker study were assayed [7]. Plasma aliquots were thawed on wet ice just prior to use, diluted 1:2 using Diluent 2, and each sample was run in duplicate by loading 50 μl of diluted plasma samples into the wells. The V-PLEX Human Pro-inflammatory Panel 1 Human Biomarker 40-Plex Kit was used. Plates were processed according to the manufacturer’s instructions and read using the MSD MESO Sector S 600 instrument. The assay data were analyzed using MSD Discovery Workbench 4.0 software and exported to an Excel spreadsheet for further analysis. Table 1 list the number of samples for each cytokine with analyte levels above the lowest levels of detection (LLOD). Cytokines with any samples below the LLOD were excluded from further analysis. The average signal covariance of standards was 3.6%, and the average calculated concentration covariance within the detection range of the assays was 4.4%. The dynamic range was 3 logs for all assays.

**Table 1:** Total number of cytokine values above the LLOD in each CRP quartile

CRP quartiles	IFN-γ	IL-1β	IL-2	IL-4	IL-6	IL-8	IL-10	IL-12p70	IL-13	TNF-α
Q1	20	7	13	20	20	20	20	20	12	20
Q2	20	8	3	10	20	20	20	14	4	20
Q3	20	5	2	12	20	20	20	16	5	20
Q4	20	9	16	20	20	20	20	20	18	20

**Fig. 1:**
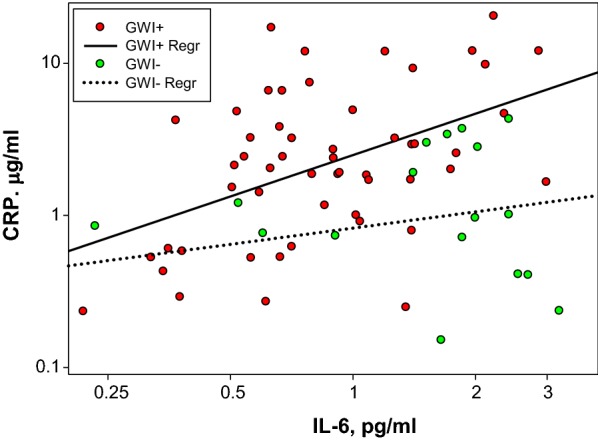
Plasma IL-6 versus Plasma CRP. The scatter plot represents the plasma IL-6 and CRP levels for each subject. The lines are the linear regression determinations for the GWI+ and GWI− groups. R^2^ values for the regressions were 0.22^GWI+^ and 0.15^GWI−^

Data calculations, Table 2, were performed using SigmaPlot (Version 11). There were no corrections for potential confounders. Data were graphed on a log_10_–log_10_ scale, Fig. 1. SigmaPlot (Version 11) was used for both the scatter plot and the linear regression calculations.

**Table 2:** Plasma biomarker distributions in GWI+ and GWI− Veterans

Biomarker	GWI+ (n = 53)	GWI− (n = 27)	P-value*
IL-6, pg/ml	0.80 (0.58–1.37)^a^	0.64 (0.50–0.93)	0.08
IFN-γ, pg/ml	2.32 (1.69–3.45)	3.32 (1.77–5.86)	0.30
IL-10, pg/ml	0.21 (0.17–0.30)	0.24 (0.18–0.36)	0.43
TNF-α, pg/ml	2.14 (1.93–2.47)	2.22 (1.92–2.58)	0.56
IL-8, pg/ml	4.43 (3.20–5.33)	3.84 (2.79–6.24)	0.94

* Mann–Whitney test

^a^Median (interquartile)

### Results

In this study, stored plasma samples from our previous GWI blood biomarker study [7] were reanalyzed using a more sensitive immunoassay system. Samples were obtained from Veterans who were deployed during the Gulf War and classified as either GWI+ or GWI− according to the CDC 10 case definition. The objective was to measure the plasma concentrations of 10 inflammation-related cytokines not detected in the previous study. To this end the MSD V-PLEX Proinflammatory Panel 1, which is sensitive to sub pg/ml cytokine concentrations, was employed.

Eighty plasma samples were assayed for 10 cytokines, and the raw signals were converted to analyte concentrations using the standard curves. For IL-β, IL-2, IL-4, IL-12 p70, and IL-13, multiple samples had concentrations that fell below the LLOD. Therefore, these cytokines were excluded from the analysis. Cytokine concentrations of IFN-γ, IL-6, IL-8, IL-10, and TNF-α were above the LLOD in all 80 plasma samples. Statistical analysis of these data is presented in Table 2. Differences in cytokine levels between the GWI + and GWI- were small. However, the GWI-associated increase in IL-6 approached statistical significance with a P-value of 0.08. The precision of the sample measurement was excellent; the average signal CV was 5.9% and the average calculated concentration CV for the samples within the detection ranges of the assays was 13.3%.

The apparent IL-6 increase in the GWI+ group prompted a closer look at the data. CRP concentrations from the previous analysis [7] were plotted versus IL-6 levels, Fig. 1. The GWI status for each data point is color coded as GWI+ (red) and GWI− (green). While there was considerable data scatter, as indicated by the linear regression R^2^ values (0.22^GWI+^, 0.15^GWI−^), CRP clearly increases as a function of IL-6 for both groups. The rate of the increase, i.e., the slope of the line (0.90^GWI+^, 0.36^GWI−^), was 2.5-fold greater for the GWI+ group.

### Discussion

An ever-increasing number of Gulf War Veterans are suffering from GWI [4–6]. Studying underlying pathophysiology is the translational path to discovery of objective diagnostic measures and evidence-based treatments. Mindful of these goals, we tested the hypothesis that the GWI-associated chronic inflammation is driven by a cascade of proinflammatory cytokine signals. Measuring plasma levels of proinflammatory cytokines may provide direct evidence of an activated inflammatory cascade in GWI.

The GWI blood biomarkers detected in our initial study [7] are all related to inflammation. Also, inflammation can cause all the symptoms of GWI [8–15]. Thus, it is our assertion that chronic inflammation is the underlying cause of GWI symptoms. The biomarker evidence supports a molecular mechanism in which GWI begins with a pro-inflammatory stimulus. Immune cells respond to the stimulus by synthesizing pro-inflammatory cytokines that initiate the inflammatory cascade [16, 17]. Next, these primary cytokines act via autocrine, paracrine, and endocrine signaling pathways to alter gene expression and produce the GWI blood biomarker fingerprint.

It is our contention that these blood-based phenotypic changes, induced by environmental toxins and stresses, are the systemic events that produce the cluster of symptoms we call GWI. In our model of GWI, there are three key steps: (1) The inflammatory cascade is triggered by toxic exposures and stresses such as those experienced by US military personnel in the Kuwaiti Theater of Operations. (2) The initial innate immune response is transformed into a chronic inflammatory state. (3) Chronic inflammation causes GWI-associated symptoms. The mechanism of the transition from an innate immune response to a chronic inflammatory state remains obscure, but this conversion is a potential high-value target for future interventions aimed at preventing chronic multisymptom illness.

The objective of the current study was to test the pro-inflammatory cytokine hypothesis. A major prediction, based on the results of the first study, is the GWI-associated increases in CRP are the result of increases in plasma IL-6 [18–20]. This objective was achieved by reanalyzing the stored plasma samples from the initial study with a more sensitive immunoassay system, the MSD-ECL platform. This system allowed us to measure pg/ml plasma concentrations of 10 cytokines, and obtain new evidence demonstrating a positive correlation between plasma levels of CRP and IL-6. This result suggests that IL-6 is a key proinflammatory signal driving the inflammatory cascade in GWI.

CRP gene expression is driven by IL-6, and IL-6 gene expression is driven by IL-1β. Our IL-1β data are limited by the fact that in 51 out of 80 samples the plasma level of IL-1β was below the LLOD. Thus, we cannot draw any direct conclusion about the role of IL-1β in GWI. The IL-6 and CRP data indicate IL-1β is involved. However, direct evidence of a causal role for IL-1β in GWI must await a more sensitive assay.

Of note, median biomarker levels for the Veterans in this study with prevalent GWI were still within standard “normal” thresholds, and thus would not have been flagged by other studies that did not include a healthy control group. It is well established that inflammation increases with older age [21, 22], a phenomenon that could contribute to cumulative inflammatory burden and rising GWI incidence as this Veteran population ages.

We have presented evidence of a correlation between increased plasma levels of IL-6 and CRP in Gulf War Veterans. The rate of increase is greater for in the GWI+ group. This result support the conclusion of our previous blood biomarker study that low-grade chronic inflammation explains the high prevalence of GWI in Gulf War Veterans.

## Limitations

- Small sample size (80 subjects).
- One-time sampling (not longitudinal).
- Small number of cytokines assayed [10].
- Complete data for only 5 of the cytokines.

## Data Availability

The datasets used and/or analysed during the current study are available from the corresponding author on reasonable request.

## Abbreviations

CDC: Centers for Disease Control and Prevention
CDC-10: CDC 10 question symptoms assessment survey
CMI: chronic multisymptom illness
CRP: C-reactive protein
GWI: Gulf War Illness
GWI+: symptomatic Gulf War Veterans
GWI-: asymptomatic Gulf War Veterans
IFN-γ: interferon gamma
IL-1β: interleukin 1 beta
IL-2: interleukin 2
IL-4: interleukin 4
IL-6: interleukin 6
IL-8: interleukin 8
IL-10: interleukin 10
IL-12: p70 interleukin 12 p70
IL-13: interleukin 13
LLOD: lowest levels of detection
MSD: Meso Scale Discovery
TNF-α: tumor necrosis factor alpha
VA: Department of Veterans Affairs
